# Expression of glucocorticoid receptor (GR) and clinical significance in adrenocortical carcinoma

**DOI:** 10.3389/fendo.2022.903824

**Published:** 2022-08-05

**Authors:** Kan Wu, Zhihong Liu, Jiayu Liang, Fuxun Zhang, Fan Zhang, Yaohui Wang, Thongher Lia, Shenzhuo Liu, Yuchun Zhu, Xiang Li

**Affiliations:** Department of Urology, Institute of Urology, West China Hospital, Sichuan University, Sichuan, China

**Keywords:** glucocorticoid receptor, adrenocortical carcinoma, immune signature, survival outcome, immunohistochemistry

## Abstract

Adrenocortical carcinoma (ACC) is a rare endocrine tumor, and most cases present with hormone excess with poor prognosis. Our research aims to determine the clinical and biological significance of glucocorticoid receptor (GR) expression using large cohorts of ACC patients. Immunohistochemistry was used to assess the expression of GR in 78 ACC cases from the West China Hospital (WCH) cohort. RNA-seq data were retrieved from The Cancer Genome Atlas database (TCGA, n=79). Clinicopathological and follow-up data were obtained from two cohorts. The correlation between the GR gene and tumor immune status was estimated using TIMER and GEPIA2. Kaplan–Meier analysis was performed to identify the prognostic value of GR in ACC. In the WCH cohort, positive nuclear GR staining was identified in 90% of the primary ACC cases. Cortisol-secreting ACCs demonstrated significantly lower GR protein expression than did nonfunctioning tumors (P<0.001). This finding was validated by the mRNA data analysis of the TCGA cohort (P = 0.030). GR expression was found to be positively correlated with the immune cell infiltration level and immune-checkpoint-related gene expression in ACC. Survival comparison and multivariate analysis showed that GR expression is an independent prognostic predictor of disease-free survival and overall survival in ACC patients in both cohorts. Our findings suggest that low GR expression is significantly correlated with excess cortisol, immune signatures and poor survival in ACC patients. We propose that GR signaling may play an important role in ACC behavior and thus may be a therapeutic target, which deserves further research.

## Introduction

Adrenocortical carcinoma (ACC) is a rare malignant tumor with an annual incidence of 0.7-2 per million population (1, 2). Although the incidence of ACC is low, the prognosis is poor, and the 5-year survival for metastatic disease is 0% to 28% (3, 4). Approximately 30% of patients with ACC may experience disease recurrence, even after margin-negative resection (5). ACCs usually present with symptoms of hormonal excess or local compression caused by abdominal mass. Approximately 50-60% of ACC patients have autonomous hormone excess, with cortisol secretion being the most common type, with up to 40% of those with Cushing syndrome or hypercortisolism combined with sex steroids (6, 7). The only curative treatment for ACC remains complete surgical resection. There are no available therapeutic regimens for recurrent or metastatic disease with unsatisfactory response.

In recent years, considerable efforts have been made to determine risk factors associated with recurrence and survival, including tumor stage, age, sex, hormone section and molecular markers (8). Additionally, multiple studies have reported that tumors with cortisol excess could influence the prognosis of patients with ACC. A previous study based on our ACC cohort also found that hypercortisolism is an independent risk factor for patient survival (9). A recent meta-analysis focused on the impact of hormonal functional status on survival in ACC and provided pooled data on 3814 patients from 19 studies (10). The meta-analysis demonstrated that cortisol-secreting ACCs had higher recurrence and mortality risk than noncortisol-secreting ACCs (10). These studies suggest that cortisol-secreting ACCs have a more aggressive clinical behavior and worse prognosis, representing an aggressive ACC subtype.

Glucocorticoids (GCs; i.e., Cortisol, as a steroid hormone, functions mostly through transcriptional regulation of glucocorticoid receptor (GR) and plays essential roles in various physiological and pathological processes, such as cell proliferation, metabolism, immune response, development and tumor biology (11). GR is ubiquitously expressed in both normal and cancer cells and has nuclear transcription factor and chromatin remodeling functions (12). The biological function of GR in metabolism and physiology is cell-type specific, and its role in regulating tumor development appears to also depend on tumor type and microenvironment. For example, extensive studies have shown that increased GR expression may be correlated with the aggressiveness and poor prognosis of many cancers, such as ovarian cancer, breast cancer and castration-resistant prostate cancer (13–15). In contrast, other studies indicated that GR loss or downregulation can be observed in other cancers, leading to malignant transformation (11).

The dual role of GR in solid tumors may also be due to cancer subtype specificity. Despite extensive studies of GR in many cancers, its role in ACC has not been determined conclusively. An earlier study only analyzed the diagnostic value of GR in ACC and discovered that GR is significantly overexpressed in ACC compared with adrenocortical adenomas (ACA)s (16). It is well known that glucocorticoids can suppress immunity and promote tumor development by regulating the level of tumor-infiltrating immune cells. Recently, Figueiredo and colleagues conducted a comprehensive silico transcriptome analysis of ACC based on public databases, and found that immune signature was associated with the steroid profiles of ACC, patients with low steroid phenotype had a higher pattern of immune activators and higher immune cell infiltration than patients with high steroid phenotype (17). This study highlighted the impact of excess steroids on the immune system interaction with ACC tumors, drawing attention to potential therapeutic targets. Given that GR is the main molecule mediating the regulation of cortisol, we speculate that GR may also be involved in the process of cortisol’s immunosuppressive effect by regulating tumor immune cell infiltration. Therefore, these observational data prompt further research on the role of GR in the oncogenesis and behavior of ACC. The purpose of this study was to evaluate GR in large and well-characterized ACC samples using immunohistochemistry to determine the relationship between GR expression and clinicopathological features and patient outcomes.

## Materials and methods

### Patient cohort

Two cohorts of ACC patients were enrolled in this study. One cohort was composed of 78 nonmetastatic ACC patients who received radical adrenalectomy at the Department of Urology, West China Hospital (WCH), Sichuan University between 2009 and 2019. Corresponding clinicopathological information and formalin-fixed, paraffin-embedded (FFPE) tissues were collected from our institution. The method and standard of clinical record collection and follow-up protocol were the same as those in our previous report (18). To further validate GR’s clinical significance, transcriptome profiles and clinical follow-up data were retrieved from the Cancer Genome Atlas (TCGA) database (including 79 ACC patients) (19). This study was approved by our institutional review board.

### Gene expression analysis

For GR mRNA expression, analysis was based on publicly available TCGA ACC tumors, and expression data were then log2(x+1) transformed. In addition, to compare GR expression levels in ACCs, ACAs and normal tissues, we downloaded the GSE10927 microarray data for analysis from the Gene-Expression Omnibus (GEO) database (http://www.ncbi.nih.gov/geo), which contains 33 ACC, 22 ACA and 10 normal adrenal cortex samples, each from a different patient, had mRNA assays performed using Affymetrix HG_U133_plus_2 arrays, with 54675 probe-sets (20). The raw data were downloaded as MINiML files and normalized by log2 transformation. The result of the data preprocessing is displayed by boxplot.

### Immunohistochemistry and evaluation

Serial FFPE tissue sections with a thickness of 5 μm were subjected to immunohistochemical staining for GR, following standard protocols we previously described (21). Briefly, sections were deparaffinized and rehydrated through xylene and ethanol, respectively. Then, tissue antigen retrieval was performed with citrate buffer solution. After being placed in 3% H2O2 and blocking buffer at room temperature, slides were incubated with anti-GR antibody (#12041; 1:400; Cell Signaling Technology) overnight at 4°C. The stained tissue slides were scanned by a digital pathology slide scanner (NanoZoomer Digital Pathology, Japan) and independently evaluated by two investigators. A modified “quick-score” protocol was used to evaluate immunostaining of GR by multiplying the staining intensity score (0: negative, 1: weak, 2: intermediate and 3: strong) by the proportion score (0 = absent, 1 ≤ 25%, 2 ≤ 50%, 3 ≤ 75%, 4 ≥ 75%). Only tumor cell staining was scored. The immunoreactivity score ranged from 0 to 12. The cut-off value for distinguishing high and low expression of GR was based on the distribution of immunoreactivity scores, i.e. the top quartile compared to the others (score = 12 vs < 12). All patients were divided into high GR (score = 12) or low/intermediate GR (score < 12) groups based on GR immunoreactivity scores in primary ACC tissue specimens. The Ki67 index retrieved from pathology records was also categorized into low (< 20%) and high (≥ 20%).

### Analysis of the immune status of ACC

Analysis of publicly available TCGA and GTEx data was performed using GEPIA2 (http://gepia2021.cancer-pku.cn/) to compare the mRNA expression of GR in CD4+ cells, CD8+ cells and macrophages in ACC and normal adrenal glands. Additionally, TIMER (https://cistrome.shinyapps.io/timer/) was used to estimate the potential correlation between GR gene expression and abundance of immune infiltrates. The correlation between the GR gene and six immune infiltrating cells (B cells, CD4+ T cells, CD8+ T cells, neutrophils, macrophages and dendritic cells) was estimated by TIMER algorithm (22). Additional xCell algorithm was applied to validate the GR expression groups in terms of immune cell infiltration (23). Further, SIGLEC15, TIGIT, CD274, HAVCR2, PDCD1, CTLA4, LAG3 and PDCD1LG2 were selected to be immune-checkpoint–relevant transcripts (24), and the expression values of these eight genes were compared in the GR-high and GR-low groups.

### Functional and pathway enrichment analysis

RNA-sequencing expression profiles and corresponding clinical information for ACC were downloaded from the TCGA dataset (https://portal.gdc.com) (19). First, we analyzed and obtained differentially expressed genes (DEGs) between the GR-low group and GR-high group using the “Limma” package. Adjusted p < 0.05 and log (fold change) > 1 or log (fold change) < -1 were defined as the thresholds for screening DEGs. To further understand the potential biological functions and signaling pathways regulated by GR genes, Gene Ontology (GO) enrichment analysis and Kyoto Encyclopedia of Gene and Genomes (KEGG) pathway enrichment analysis were performed by using the “ClusterProfiler” package. The GO enrichment analysis, including molecular function, cellular component and biological process, was plotted by using Goplot. P < 0.05 or false discovery rate (FDR) < 0.05 was considered the cutoff criterion for meaningful pathways.

### Statistical analyses

The clinicopathological and biological variables between groups were compared by using the chi-square test or Fisher’s exact test. Correlations between different variables were examined using Pearson’s chi-squared test. In the TCGA cohort, ACC patients were classified based on GR mRNA expression being in the top quartile of expression versus all other patients. Similarly, in the WCH cohort, ACC patients were classified according to GR immunoreactivity score being in the top quartile of score versus all others. The cutoff values for survival comparisons were determined based on all patients in a given group of each cohort. Overall survival (OS), cancer-specific survival (CSS) and disease-free survival (DFS) were estimated using Kaplan–Meier analysis and compared between the high GR expression group (top quartile) and the low GR expression group by the log-rank test. A Cox proportional hazards regression model was used to determine independent factors associated with survival. Hazard ratios (HRs) with 95% confidence intervals (CIs) were generated by Cox regression. All statistical analyses were performed using R software (version v4.0.3, the R Foundation for Statistical Computing, 2020) and Prism (V.5.0a, GraphPad Software). P < 0.05 was considered significant.

## Results

### Protein expression of GR in ACC primary tumors

**Figure 1:**
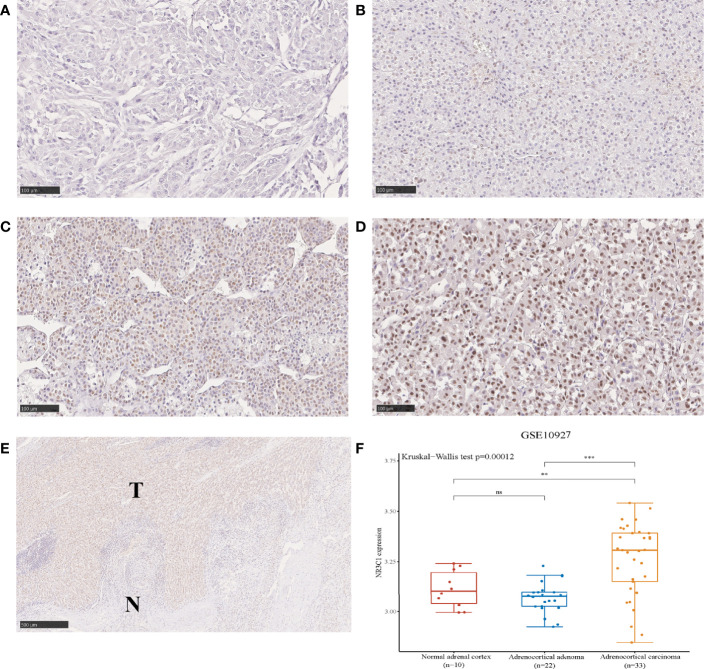
The protein expression of GR in ACC showing negative **(A)**, weak **(B)**, intermediate **(C)** and strong **(D)** intensities of GR expression in carcinomas (100 µm). Immunohistochemical staining shows the higher expression of GR in tumor tissues compared to adjacent normal tissues **(E)**. The expression distribution of GR gene in normal adrenal cortex, ACA and ACC **(F)**. *p < 0.05, **p < 0.01, ***p < 0.001; ns, no significance.

The different patterns of GR protein expression in ACC primary tumors are shown in **Figure 1**. The expression of GR in the nucleus of tumor cells was evident but sparsely stained in the cytoplasm. The median immunoreactivity score was 6 (range 0-12). Out of 78 cases in the WCH cohort, 70 (90%) were GR positive in the nucleus. The interobserver agreement for the classification of GR staining was very good, with a Cohen k coefficient of 0.88. GR was found to be over-expressed in ACC tissues compared to that in normal tissues (**Figure 1E**). In addition, based on the microarray data (GSE10927) in ACCs (n = 33), ACAs (n = 22) and normal adrenal cortex (n = 10), GR was overexpressed in ACCs compared with ACAs and normal tissues (**Figure 1F**).

### Relationship between GR expression and clinicopathological and biological features

**Figure 2:**
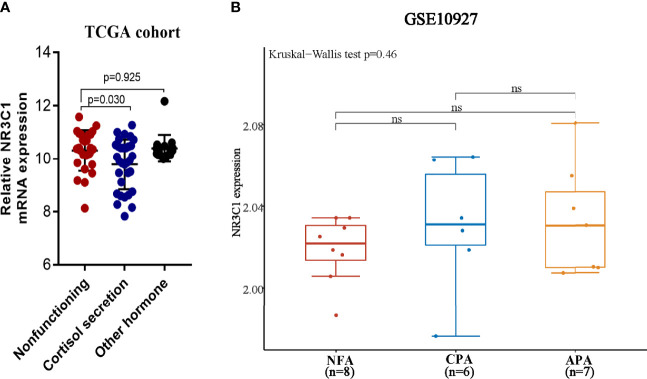
The mRNA expression of GR in the ACC and adrenal adenoma. **(A)** Relative mRNA expression of GR in the nonfunctioning tumors, cortisol-secreting ACC and other hormone-secreting ACC. **(B)** Relative mRNA expression of GR in nonfunctional adenoma (NFA), cortisol-producing adenoma (CPA) and aldosterone-producing adenoma (APA). ns, no significance.

The relationship between GR and clinicopathological and biological characteristics is shown in **Table 1**. The clinicopathological parameters (age, sex, tumor stage, Weiss score and Ki67 index) were equally distributed between the GR-high and GR-low groups, except for the hormonal hypersecretion feature, which was higher in the GR-low group in the WCH (73% vs. 26%, p < 0.001) and TCGA (72% vs. 41%, p = 0.020) cohorts. The RNA-sequencing data analysis of TCGA ACC cohort confirmed GR mRNA expression to be significantly downregulated in cortisol-secreting ACCs compared with clinically nonfunctioning tumors (p = 0.030, **Figure 2A**). However, the mRNA expression of GR did not differ between other secreting and nonfunctioning tumors and other functional ACCs (p = 0.925, **Figure 2A**), suggesting that this phenomenon may be caused by specific secretion. In addition, we analyzed GR expression in nonfunctioning versus cortisol-secreting adrenal adenomas as well as aldosterone-producing adenomas to determine whether the decrease was due to general negative feedback. There was no difference in the expression of GR among the three types (p = 0.61, **Figure 2B**).

**Table 1:** Clinicopathological characteristics of ACC patients in WCH and TCGA cohort.

	WCH cohort (n=78)				TCGA cohort (n=79)			
		GR expression				GR expression		
Characteristics	Patients, n (%)	Low (N=55)	High (N=23)	P value	Patients, n (%)	Low (N=59)	High (N=20)	P value
Age (y)				0.208				0.948
< 50	49 (63%)	37 (67%)	12 (52%)		40 (51%)	30 (51%)	10 (50%)	
≥50	29 (37%)	18 (33%)	11 (48%)		39 (49%)	29 (49%)	10 (50%)	
Gender				0.713				0.542
Male	33 (42%)	24 (44%)	9 (39%)		31 (39%)	22 (37%)	9 (45%)	
Female	45 (58%)	31 (56%)	14 (61%)		48 (61%)	37 (63%)	11 (55%)	
ENSAT stage				0.670				0.153
Low (I, II)	55 (71%)	38 (69%)	17 (74%)		46 (60%)	32 (55%)	14 (74%)	
High (III, IV)	23 (29%)	17 (31%)	6 (26%)		31 (40%)	26 (45%)	5 (26%)	
Laterality				0.111				0.076
Left	31 (40%)	25 (46%)	6 (26%)		45 (57%)	37 (63%)	8 (40%)	
Right	47 (60%)	30 (54%)	17 (74%)		34 (43%)	22 (37%)	12 (60%)	
Hormone secretion excess				**< 0.001**				**0.020**
No	32 (41%)	15 (27%)	17 (74%)		26 (35%)	16 (28%)	10 (59%)	
Yes	46 (59%)	40 (73%)	6 (26%)		48 (65%)	41 (72%)	7 (41%)	
Ki67 index				0.964				0.271
Low	41 (53%)	29 (53%)	12 (52%)		39 (49%)	27 (46%)	12 (60%)	
High	37 (47%)	26 (47%)	11 (48%)		40 (51%)	32 (54%)	8 (40%)	

ACC, adrenocortical Carcinoma; WCH, West China Hospital; TCGA, The Cancer Genome Atlas; ENSAT, European Network for the Study of Adrenal Tumors. The bold value represent statistical significance (P < 0.05).

**Figure 3:**
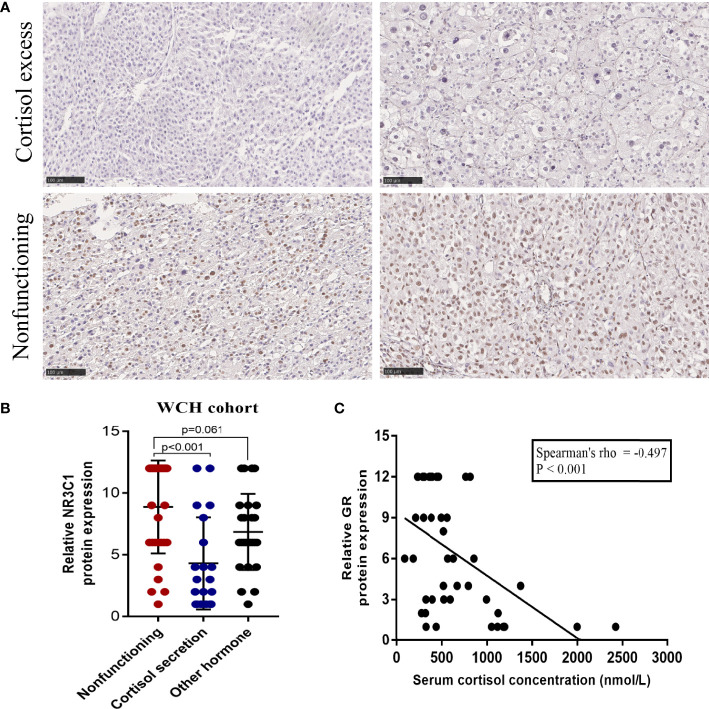
The protein expression of GR is downregulated in the cortisol-secreting ACC. **(A)** Representative immunohistochemical staining of GR in the nonfunctioning tumors and cortisol-secreting ACCs (100 um). **(B)** Protein expression of GR in nonfunctioning tumors, cortisol-secreting ACC and other hormone-secreting ACC. **(C)** Correlation analysis between GR expression and serum cortisol levels in patients with ACC in the WCH cohort.

In the WCH cohort, as expected, intense GR staining was observed in nonsecreting tumors, while low staining was observed in cortisol-producing tumors (**Figure 3A**). Consistently, cortisol-secreting ACCs demonstrated significantly lower GR protein expression than nonfunctioning tumors (p < 0.001, **Figure 3B**), and no differences in GR expression between nonfunctioning tumors and other hormone-secreting tumors were observed (p = 0.061, **Figure 3B**). Furthermore, we found that the protein expression of GR was inversely correlated with serum cortisol levels in ACC patients (Spearman’s rho = -0.497, p < 0.001; **Figure 3C**). These results revealed that in ACC, GR expression was lower in cortisol-secreting tumors at both the RNA and protein levels.

### Influence of GR expression on the immune status of ACC

**Figure 4:**
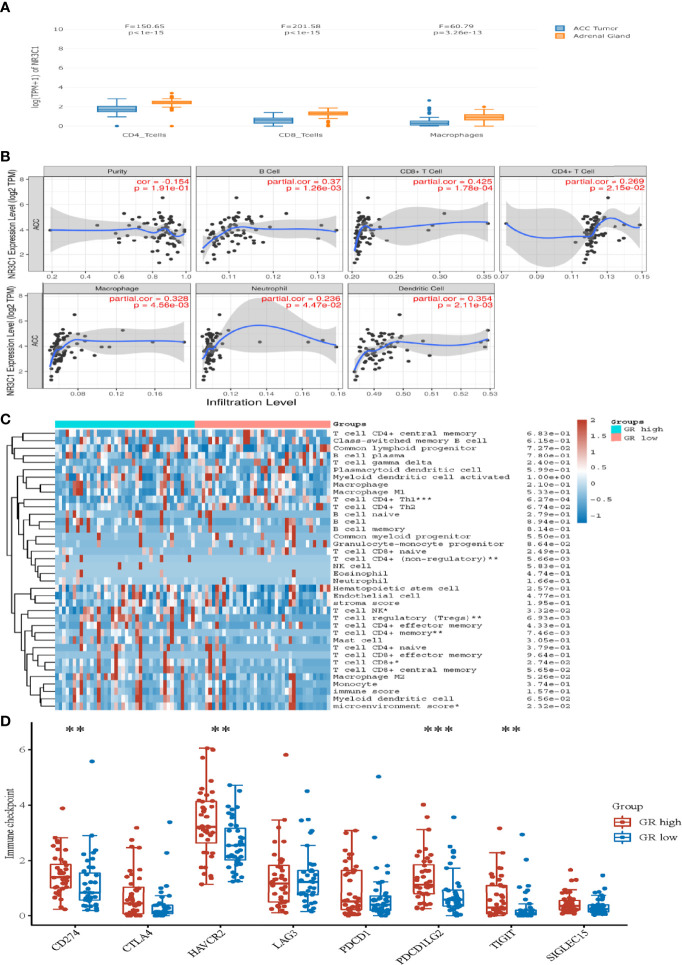
The influence of GR mRNA expression on the immune status of ACC. **(A)** Comparison of GR mRNA expression between CD4+ T cells, CD8+ T cells and macrophages in ACC and normal adrenal glands using publicly available datasets. (GEPIA2021, subexpression analysis, http://gepia2021.cancer-pku.cn/). **(B)** The correlation between immune cell infiltration and mRNA expression levels of GR in ACC tumors. (TIMER, https://cistrome.shinyapps.io/timer/). **(C)** Immune cell score heatmap shows the expression distribution of immune score in GR-low ACC and GR-high ACC. **(D)** The expression distribution of immune checkpoints gene in GR-low ACC and GR-high ACC.

We subsequently analyzed TCGA-ACC data using GEPIA2021 and performed a cell type-level expression analysis with the EPIC algorithm to examine the expression pattern of GR mRNA in CD4+, CD8+, and macrophages in ACC and adrenal glands. GR mRNA levels were significantly lower in CD4+, CD8+, and macrophages of ACC than in normal adrenal tissue (**Figure 4A**). To explore the potential correlation of GR with the immune microenvironment in ACC, the relationships between GR and the levels of immune cell infiltration in tumors were evaluated by using TIMER. GR expression was found to be positively correlated with the infiltration signatures of B cells, CD8+ T cells, CD4+ T cells, macrophages, neutrophils and dendritic cells (**Figure 4B**). The immune cell score for the xCell results based on TCGA ACC patients are presented in **Figure 4C**. Consistent with the TIMER analysis, the xCell results also showed that high GR tumors were significantly associated with high infiltration of CD4+ T-cells (p = 0.00566), NK cells (p = 0.0332), T cell regulatory (p = 0.0093), and CD8+ T (p = 0.0274). Which suggest a higher immune cell presence in patients with GR-high ACC compared to those in patients with GR-low ACC. On the other hand, we did not see any association of B cells, dendritic cells, macrophages (M1 or M2), neutrophils, mast cells, and monocytes infiltration with GR expression (**Figure 4C**). In addition, the expression distribution of immune checkpoints gene in two groups are presented in **Figure 4D**. GR-high ACC was rich in CD271 (PD-L1), HAVCR2, PDCD1LG2 (PD-L2) and TIGIT (the Wilcox test: p < 0.01, p < 0.01, p < 0.001, P < 0.01), suggesting patients with GR-high had higher expression of immune modulators than patients with GR-low ACC. While, we observed no difference in the expression of CTLA4, LAG3, PDCD1 and SIGIEC15 between the two groups (**Figure 4D**).

### Impact of GR expression on disease-free survival and overall survival

**Figure 5:**
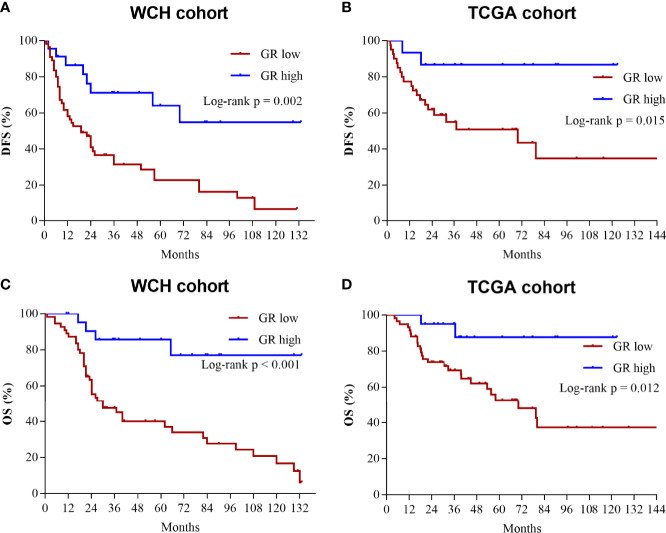
Low levels of GR predict worse prognosis in ACC patients. **(A)** Cumulative overall survival curves of patients from the WCH cohort with high or low GR expression levels. **(B)** Cumulative disease-free survival curves of patients from the WCH cohort with high or low GR expression levels. **(C)** Cumulative overall survival curves of patients from the TCGA cohort with high or low GR expression levels. **(D)** Cumulative disease-free survival curves of patients from the TCGA cohort with high or low GR expression levels.

The survival outcomes of GR expression in the WCH and TCGA cohorts are shown in **Figure 5**. Patients with GR-low tumors had a higher risk of recurrence or metastasis (5-DFS rates: 23% vs 64%, p = 0.002; **Figure 5A**) and worse survival outcomes (5-OS rates: 40% vs 86%, p < 0.001; **Figure 5C**) in WCH. This survival difference was confirmed in the TCGA cohort, and low GR expression was also strongly correlated with poor DFS (5-DFS rates: 51% vs 87%, p = 0.015; **Figure 5B**) and OS (5-OS rates: 53% vs 88%, p = 0.012; **Figure 5D**) in ACC patients. Moreover, the multivariate Cox regression model (**Table 2**) demonstrated that GR expression had independent prognostic value for CSS and OS in the WCH and TCGA cohorts, taking into account other prognostic factors (including patient age, sex, tumor stage, resection status, glucocorticoid excess and Ki67 index). Together, these results suggest that GR expression levels can stratify most ACC patients.

**Table 2:** Cox multivariate regression analyses of parameters associated with survival in WCH and TCGA cohort.

Variables	WCH			TCGA		
	HR	95% CI	P Value	HR	95% CI	P Value
**OS**						
Age	1.23	0.62-2.43	0.554	1.92	0.76-4.87	0.167
≥50 vs >50 years						
Gender	1.15	0.61-2.17	0.672	2.06	0.71-5.97	0.182
Female vs male						
Tumor stage	1.07	0.53-2.18	0.846	1.15	0.41-3.26	0.795
I/II vs III/IV						
Resection status	1.98	1.01-3.86	**0.046**	9.01	1.39-58.55	**0.021**
R>0 vs R=0						
Ki67 index	2.57	1.29-5.13	**0.007**	4.87	1.37-17.30	**0.014**
High vs low						
Cortisol excess	2.12	1.10-4.10	**0.025**	2.69	1.05-6.87	**0.039**
Yes vs no						
GR	0.26	0.09-0.78	**0.016**	0.20	0.04-0.93	**0.041**
High vs low						
**CSS**						
Age	1.19	0.64-2.21	0.587	1.98	0.77-5.08	0.155
≥50 vs >50 years						
Gender	1.04	0.57-1.86	0.909	1.67	0.56-5.00	0.361
Female vs male						
Tumor stage	1.18	0.63-2.22	0.601	1.17	0.40-3.45	0.781
I/II vs III/IV						
Resection status	2.08	1.11-3.90	**0.022**	8.97	1.25-64.56	**0.029**
R>0 vs R=0						
Ki67 index	2.47	1.29-4.75	**0.007**	4.39	1.25-15.41	**0.021**
High vs low						
Cortisol excess	1.80	1.00-3.25	**0.050**	3.05	1.14-8.17	**0.027**
Yes vs no						
GR	0.43	0.19-0.98	**0.045**	0.10	0.01-0.78	**0.028**
High vs low						

WCH, West China Hospital; TCGA, The Cancer Genome Atlas; HR, hazard ratio; CI, confidence interval; OS, overall survival; CSS, cancer-specific survival. The bold value represent statistical significance (P < 0.05).

### GR expression and associated biological processes

To further explore the biological actions of GR genein ACC. First, we identified 295 genes differentially expressed between the GR-low group and GR-high group. Among these genes, there were 121 upregulated genes and 174 downregulated genes in the GR-low group (**Supplementary Figures A, B**). Furthermore, KEGG pathway (Up) enrichment analysis revealed that the overexpressed genes were mainly enriched in “cAMP signaling pathway”, “Cortisol synthesis and secretion”, “Cushing syndrome”, and “Aldosterone synthesis and secretion” (**Supplementary Figure C**). The GO (Up) enrichment analysis revealed that overexpressed genes were significantly enriched in “steroid biosynthetic and metabolic process”, “hormone metabolic process” and “sterol biosynthetic and metabolic process” (**Supplementary Figure D**). Altogether, the enrichment analysis of upregulated genes indicated that steroid biosynthetic and metabolic-related processes were activated in GR low ACCs. After that, we also performed the KEGG pathway (Down) enrichment analysis, which showed that the down-regulated genes were remarkably enriched in “T helper cell differentiation”, “JAK-STAT signaling pathway”, and “regulation of cell–cell adhesion” (**Supplementary Figures C, D**).

## Discussion

In this study, GR expression was demonstrated in 90% of primary ACC cases and up-regulated in tumor tissues compared to adjacent normal tissues, which is in agreement with previous findings (16). Within the ACCs, strong GR staining was observed in a higher proportion of nonfunctioning tumors compared to hormonal functioning ACCs, while some cortisol-secreting tumors even did not express this receptor. As expected, we confirmed that GR gene is an independent predictor of DFS and OS in patients with ACC. Therefore, GR expression profiles in ACC indicate that GR would be considered a prognostic and biological biomarker to stratify patients into low- or high-risk subgroups to provide rational follow-up procedures and treatment stratification based on patients’ GR signature. Further functional studies should be encouraged to better investigate the role of GR in ACC tumorigenesis and immune signatures to identify new therapeutic targets.

Several molecular mechanisms of ligand-mediated homologous downregulation of GR have been extensively described, specifically including transcriptional repression, posttranslational protein degradation and the stability of mRNA levels (25). At the RNA level, several studies have found that the mRNA levels of GR are reduced by 50-80% in many different tissues when treated with GCs (26). This phenomenon is mediated by the inhibition of transcription requiring an intragenic element consisting of the GR gene itself (27). Additionally, earlier studies have shown that GC treatment significantly decreases GR protein stability and GR protein half-life, while proteasome inhibitors can abolish GC-induced GR protein downregulation (26). Therefore, the ubiquitin–proteasome system is an important mechanism for ligand-dependent GR protein degradation. Recently, several microRNAs upregulated by glucocorticoids were shown to repress GR in human adipogenesis by reducing the stability of GR mRNA levels (28, 29). Overall, multiple mechanisms are involved in the reduction of GR levels induced by GCs. Likewise, observations in ACC samples also indicated that GCs can significantly downregulate GR at both the mRNA and protein levels.

There are conflicting results on the effects of GR on cancer cells, which range from promoting cancer progression to suppressing tumor cell growth. However, the role of GR in ACC is still debated and even unknown. A previous study assessed the diagnostic utility of GR in ACC cases displaying borderline histology, but they failed to show its prognostic role in ACC due to limited samples (16). In the present study, we found that low GR expression was associated with excess cortisol, higher recurrence risk and worse survival. The interpretation of this observation is not straightforward, and the finding cannot formally establish causality. A higher mortality risk may be explained by multisystem effects of hypercortisolism, including cardiovascular risks, peptic ulcers, fractures, and infections (30). However, the higher risk of recurrence cannot be convincingly explained by excess cortisol. One explanation is that GR signaling plays an important functional role in ACC behavior. Future research should focus on investigating whether GR signaling affects tumor cell biology or whether GR is merely a prognostic indicator.

The interplay between cortisol production and the immune status in ACC has been a topic of recent studies (17, 31). One study based on TCGA dataset analysis found that ACC patients with low steroid phenotype had higher expression of immune checkpoints gene and immune cell infiltration than patients with high steroid phenotype (17). Moreover, Landwehr and colleagues performed immunofluorescence analysis to visualize tumor-infiltrating T cells in 146 ACC tissue, and demonstrated that glucocorticoid excess is associated with T cell depletion and unfavorable prognosis in ACC (31). Given that cortisol acts primarily through transcriptional regulation of GR, we hypothesized that GR may be a key gene mediating the immunomodulatory effects of cortisol. In this study, we observed low GR expression was detected in cortisol-secreting ACCs. Remarkably, our analysis revealed that GR expression was related to the level of immune cell infiltration and immunomodulatory expression in ACC. To our knowledge, this is the first study to find significant differences in the immune cell landscape in the GR-high vs. GR-low ACC. In order to understand the potential mechanism of GR regulating the immune microenvironment state of ACC, we performed KEGG pathway enrichment analysis, and found that the JAK-STAT signaling pathway was significantly down-regulated in the GR low subgroup. However, the JAK-STAT signaling is closely related to tumorigenesis and abnormal immune surveillance, and is a major regulatory pathway for immune cell development, maturation, survival, and function (32, 33). For example, Binding of interferon to its receptor stimulates the phosphorylation of JAK1 and JAK2, which in turn phosphorylates STAT1. Phosphorylated STAT1 translocates into the nucleus, binds to the promoter element of IRF1 and drives its transcription, thereby increasing the expression of major histocompatibility class (MHC) molecules (32). Deletion or downregulation of components of the JAK pathway can lead to tumor cells evading recognition by immune cells. On the other hand, the JAK signaling pathway also mediates cytokine regulation of immune cell proliferation, differentiation, and maturation (34). Overall, we uncovered more interesting findings that may guide selection criteria for ACC-targeted immunotherapy.

Because GR, as a transcription factor, is likely to directly regulate thousands of target genes that coordinately control tumor aggressiveness, it is challenging to identify the molecular mechanisms of GR that are most relevant to cancer cell survival. At present, a variety of approaches have been used to explore the molecular mechanisms of GR activation on target genes, including oligonucleotide microarray analysis (35), chromatin immunoprecipitation scanning (36), and modulation of GR signaling using specific GR modulators (37–39), siRNA (40), and genetic mutants (41). Significantly, these studies revealed that a network of GR-mediated genes may be a better index of GR activity signature than GR expression alone. Given the notable feature of cortisol secretion in ACC, understanding the GR activity signature is essential for paving the way for future therapeutic progress.

Our study has some limitations. First, this study is limited by its retrospective nature. Second, due to the limited frozen ACC samples available, we did not perform additional experiments to validate the protein and mRNA expression of GR in the WCH ACC cohort. Finally, no functional experiments were performed to investigate the impact of GR on ACC cell biology. The underlying mechanism of GR in ACC malignancy is unclear and deserves further study.

## Conclusions

In summary, it is important to fully understand the GR signature and its role in ACC. Our study shows that low GR expression is significantly correlated with excess cortisol, immune status, increased recurrence and worse outcome. Additional research efforts exploring the potential mechanism of GR signaling in the tumorigenesis and behavior of ACC are urgently needed, adding a new dimension to the future study and treatment progress of ACC.

## Data availability statement

The raw data supporting the conclusions of this article will be made available by the authors, without undue reservation.

## Ethics statement

The studies involving human participants were reviewed and approved by the Ethics Committee of West China Hospital, Sichuan University. The patients/participants provided their written informed consent to participate in this study. Written informed consent was obtained from the individual(s) for the publication of any potentially identifiable images or data included in this article.

## Author contributions

KW and ZL: study concept and design, analysis and interpretation, drafting of the manuscript, writing—review and editing, and final approval of the version to be published. JL and FXZ: substantial contributions to data acquisition. FZ and YW: data curation. KW and TL: data interpretation. SL, YZ and XL: substantial contributions to critical revision of the manuscript and final approval of the version to be published. KW and XL: study concept and design, supervision, writing—review and editing, final approval of the version to be published, agreement with all aspects of the work, and funding acquisition. All authors: final approval of the version to be published and agreement with all aspects of the work. All authors contributed to the article and approved the submitted version.

## Funding

This work was supported by the National Natural Science Foundation of China (Reference Number: 81672552), the Science and Technology Foundation of Sichuan Province (2017JY0226), the China Postdoctoral Science Foundation (2021M692281), and the PostDoctor Research Project, West China Hospital, Sichuan University (21HXBH028).

## Conflict of interest

The authors declare that the research was conducted in the absence of any commercial or financial relationships that could be construed as a potential conflict of interest.

## Publisher’s note

All claims expressed in this article are solely those of the authors and do not necessarily represent those of their affiliated organizations, or those of the publisher, the editors and the reviewers. Any product that may be evaluated in this article, or claim that may be made by its manufacturer, is not guaranteed or endorsed by the publisher.

## Supplementary material

Supplementary Figure Differential genes and functional enrichment between GR-low and GR-high subgroups. **(A)** Volcano plots and **(B)** hierarchical clustering analysis of mRNAs that were differentially expressed between GR-high tumors and GR-low tumors. **(C)** KEGG pathway enrichment analysis and **(D)** GO enrichment analysis of genes upregulated in the GR-low subgroup and genes upregulated in the GR-high subgroup.
