# Supplementary figures and images for: Expression of glucocorticoid receptor (GR) and clinical significance in adrenocortical carcinoma
Source: Front Endocrinol (Lausanne). 2022 Aug 5;13:903824. doi: 10.3389/fendo.2022.903824 (PMC9389328; doi:10.3389/fendo.2022.903824)

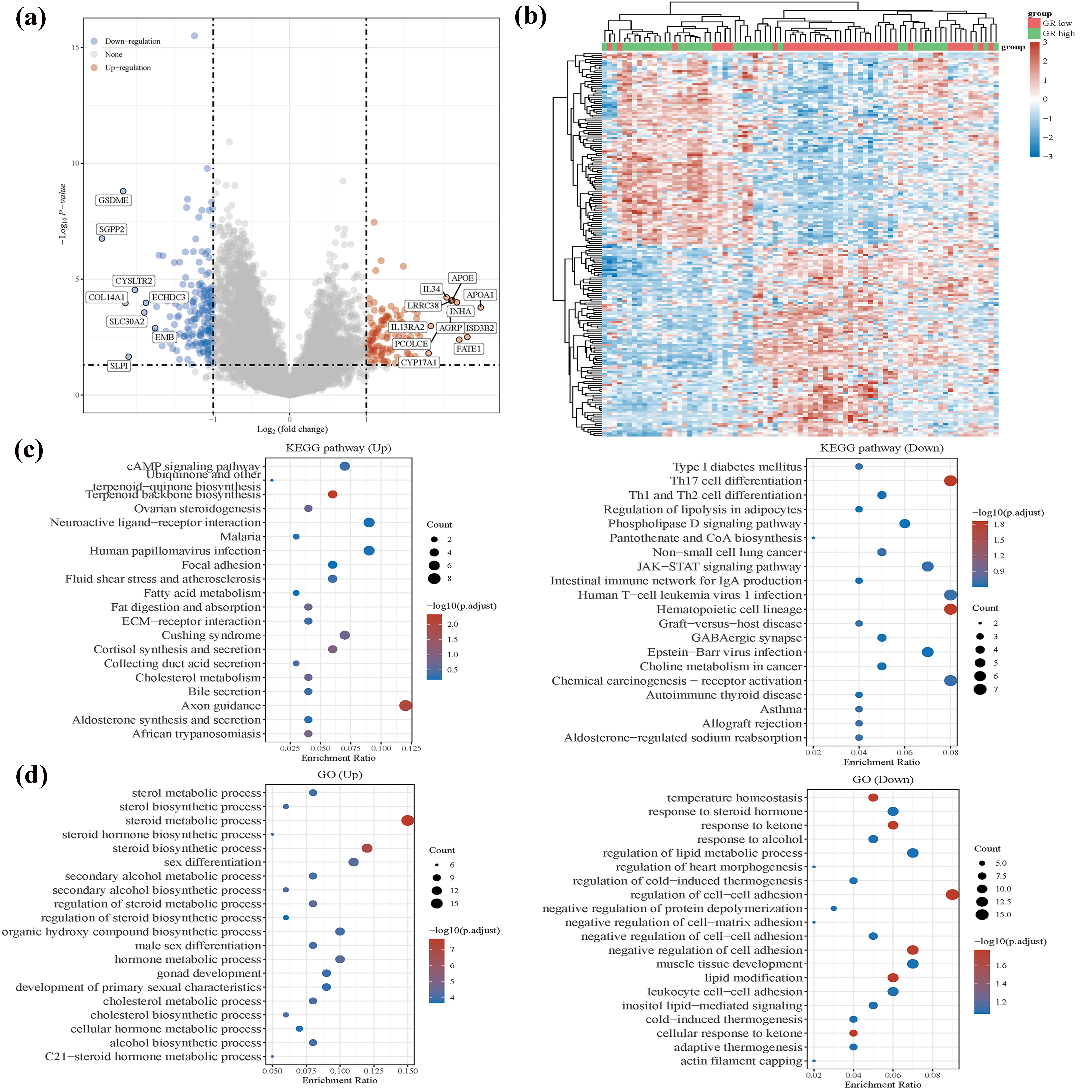

Supplement: Supplementary Figure — Differential genes and functional enrichment between GR-low and GR-high subgroups. (A) Volcano plots and (B) hierarchical clustering analysis of mRNAs that were differentially expressed between GR-high tumors and GR-low tumors. (C) KEGG pathway enrichment analysis and (D) GO enrichment analysis of genes upregulated in the GR-low subgroup and genes upregulated in the GR-high subgroup. [file Image_1.tif]
